# Combining Small-Angle X‑ray Scattering with Photoelectron Spectroscopy to Increase the Accuracy of Silica Shell Thickness Measurements on Inorganic Nanocrystals

**DOI:** 10.1021/acs.jpclett.6c02495

**Published:** 2026-09-02

**Authors:** Sarah-Luise Abram, Anna Matiushkina, David Cant, Nicholas Engel, Robin Schürmann, Jörg Radnik, Elina Andresen, Carsten Prinz, Christian Gollwitzer, Alexander Gordon Shard, Ute Resch-Genger

**Affiliations:** † Federal Institute for Material Research and Testing (BAM), Division 1.2 Biophotonics, Richard-Willstaetter Str. 11, 12489 Berlin, Germany; ‡ Free University Berlin, Department of Biology, Chemistry, and Pharmacy, Arnimallee 22, 14195 Berlin, Germany; § National Physical Laboratory (NPL), Hampton Road, TW11 0LW Teddington, U.K.; ∥ Physikalisch-Technische Bundesanstalt (PTB), Abbestr. 2-12, 10587 Berlin, Germany; ⊥ Federal Institute for Material Research and Testing (BAM), Division 6.1 Surface Analysis and Interfacial Chemistry, Unter den Eichen 44-46, 12203 Berlin, Germany

## Abstract

X-ray photoelectron spectroscopy (XPS) is widely used to measure the surface chemistry of nanomaterials, yet it can also provide structural information. The thickness of surface passivation shells can be determined with XPS and hard X-ray photoelectron spectroscopy (HAXPES), but accuracy depends upon knowledge of the density of core and shell materials. This property is typically sourced from literature data on bulk materials, which may not be correct for nanomaterials. We representatively refine shell thickness XPS and HAXPES measurements for silica shells on iron oxide and lanthanide nanoparticles, exploiting electron densities experimentally derived by small-angle X-ray scattering (SAXS) measurements of the core–shell nanoparticles in liquid mixtures with different densities. This results in improved agreement between XPS results and transmission electron microscopy (TEM) and SAXS shell thickness measurements. Thereby, HAXPES provided results in closer agreement with TEM and SAXS than XPS, likely due to a lower sensitivity to coating thickness variations.

## Graphical abstract

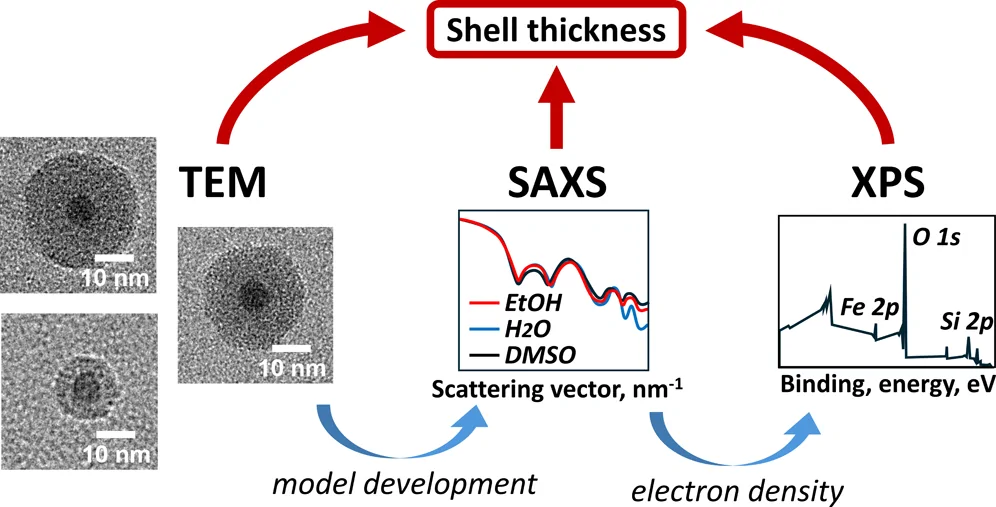

Increased use of nanomaterials in life and material sciences and consumer products necessitates reliable and accurate characterization of their physicochemical properties to ensure suitability for their intended application, including the consideration of stability, safety, and toxicity requirements.^1^ Many functional nanomaterials, utilized in the life and material sciences, such as semiconductor quantum dots,^2^ lanthanide nanoparticles,^3^ or magnetic nanoparticles,^4^ consist of core–shell structures. Typically, the surface of the core material, which possesses the desired optical, magnetic, electronic, or other functional properties, is coated with shells made from a different nanomaterial that protect the core from degradation, passivate the surface, ensure dispersion stability, and provide anchoring points for further functionalization.^5^ The chemistry, thickness, homogeneity, and morphology (including the porosity) of such shells are critical parameters that govern the performance of the core–shell particles, their stability, and interactions with their environment. Such functional requirements make reliable shell thickness measurements important for the quality control and reproducible fabrication of core–shell nanomaterials.

The dimensional characterization of core–shell nanomaterials is commonly done with transmission electron microscopy (TEM), providing information on particle shapes, size distributions, and the homogeneity of particle coatings. TEM measurements were performed on isolated particles deposited on solid supports. However, the individual evaluation of a sufficient number of representative core–shell nanoparticles is very time-consuming, and the preparation procedure may influence the representativeness of the analyzed population. Currently, emerging AI-based tools for automated electron micrograph analysis might eventually circumvent this challenge, however, their accuracy and applicability to agglomerated samples need thorough validation.^6^ Additionally, small-angle X-ray scattering (SAXS) using form-factor fitting can be employed to improve the representativeness of the dimensional measurements.^7^ This ensemble technique measures a statistically relevant number of particles in dispersion, but data evaluation is challenging, especially for nonspherical particle shapes and broad size distributions. For TEM and SAXS, an important prerequisite for a reliable shell thickness determination is a sufficient electron density contrast between the core and shell materials with SAXS additionally requiring a contrast between the shell and the surrounding liquid. Electron density contrast refers to the variations in electron or X-ray scattering within the sample, which account for the image contrast in electron micrographs and determine the scattering features in SAXS curves.

Neither of TEM nor SAXS directly provide chemical information about the core or the shell of nanomaterials. Accurate chemical analysis of such systems, with focus on coatings and surface chemistry, is typically performed with X-ray photoelectron spectroscopy (XPS), which provides information on constituting elements and their chemical state. XPS can also be exploited to measure film thickness on topographic samples, including surface-functionalized nanoparticles, and core–shell nanostructures.^8,9^ Measurements of surface coatings with thicknesses up to approximately 10 nm on flat surfaces can be achieved using traditional Al Kα X-ray sources.^10^ For core–shell nanoparticles, the information depth is lower and depends on the size and composition of the particle cores. The information depth can be increased by using hard X-ray photoelectron spectroscopy (HAXPES). For Ag Lα X-ray sources, coating thicknesses up to 20 nm may be measured on flat surfaces due to the larger inelastic mean free path (IMFP) of higher energy photoelectrons—typically more than double that of photoelectrons excited using Al Kα sources. Employing Al Kα XPS and Ag Lα HAXPES can yield information on sample composition and structure by combining information from different information depths. A comparison of thickness estimates individually obtained from XPS and HAXPES data can also allow to qualitatively assess structural features such as coating inhomogeneity. Similarly to TEM, XPS samples are analyzed in dried state deposited on a substrate. However, XPS measures large nanoparticle ensembles and does not require well separated single nanoparticles. The differentiation between core and shell material is not determined by the electron density contrast, but by the element-specific binding energies of emitted photoelectrons from the core and shell elements. A distinction is even possible when the same elements are present in both the core and the shell, but in different chemical environments. Prerequisites for the accurate calculation of the shell thicknesses from the core/shell photoelectron yield ratio by XPS are (i) concentric core–shell nanoparticles with a defined interface between core and shell and regular boundaries as well as (ii) the knowledge of the mass densities for the determination of the IMFP, to accurately consider the attenuation of the photoelectrons. Typically, literature values are used that correspond to the respective bulk materials. At the nanoscale, however, the mass density of a material can differ significantly from that of the corresponding bulk material. Also, the mass density can depend on the synthesis method used. Experimental values for nanomaterials are scarce because mass density measurements are very challenging for nanoscale systems,^11^ and are missing for core–shell nanostructures since most techniques, such as centrifugation, cannot provide separate values for core and shell materials.

According to eq 1, mass density (ρ) is directly proportional to electron density (ρ_*e*_). In eq 1, *N*_*A*_ presents Avogadro’s number, *Z* the total number of electrons, *M* the molar mass, and ρ_*e*_ the number of electrons per unit volume. The latter determines how electrons or photons are scattered or absorbed by different regions of a sample.

1 $$\rho_{e}=\frac{N_{A}\cdot Z\cdot \rho }{M}$$

An elegant approach to determine the electron density of a nanoscale shell is a contrast variation SAXS experiment,^12^ whereby the core–shell nanoparticles are measured in liquids of different electron densities to match that of the particle components. The density of liquids that retain a stable particle dispersion is, however, typically limited, and high-density materials cannot be matched by the dispersant. In this study, we utilize a modified contrast variation SAXS approach for the experimental determination of nanoparticle shell electron densities, relying on the simultaneous analysis of many scattering curves with varying contrast. These experimentally derived electron density values are then used as input for the data analysis of XPS and HAXPES measurements of core–shell nanoparticles. To demonstrate the feasibility of this approach and the effect of refining the XPS/HAXPES data analysis with the experimentally corrected values, we chose two types of widely used functional inorganic nanocrystals as core materials: (superpara)­magnetic iron oxide (IONPs) and luminescent lanthanide nanoparticles, here spectrally shifting upconversion nanoparticles (UCNPs). These nanocrystals are typically synthesized in hydrophobic solvents at high temperature to achieve high crystallinity for optimum magnetic and optical properties.^13^ To render such nanocrystals hydrophilic for life sciences applications such as bioimaging and protecting them from dissolution or aging, IONPs and UCNPs can be coated with thin silica shells.^14^ Silica shells are some of the most popular coating materials, they can be prepared straightforwardly, and are sufficiently stable and biocompatible. Also, they can be further functionalized using established silane chemistry to introduce recognition moieties, dyes or sensor molecules. Additionally, these silica shells offer a moderate electron density contrast between core and shell and do not lead to core–shell intermixing, which can occur with other surface passivation shells.^15^ To demonstrate the advantages of a combined analysis with complementary techniques, a set of IONP and UCNP core–shell particles were prepared by coating IONP and UCNP cores with silica shells of different thicknesses varying in steps of a few nm. Aiming for comprehensive and accurate measurements of the silica shell thicknesses, these samples were then analyzed with TEM, SAXS, and XPS/HAXPES, and the data were compared.

Figure 1 provides a schematic overview of this study. Monodisperse UCNP and IONP were coated with thin silica shells by a previously reported reverse microemulsion approach.^16^ Variation of the amounts of silica precursor and ammonia gave access to three different shell thicknesses per core material. TEM was used to evaluate the homogeneity and thickness of the silica shells prior to a detailed characterization by SAXS and XPS. A SAXS contrast variation experiment was performed for two representative samples to estimate the electron density of the synthesized silica shells. Subsequently, these values were used for calculating the shell thicknesses from XPS measurements.

**Figure 1:**
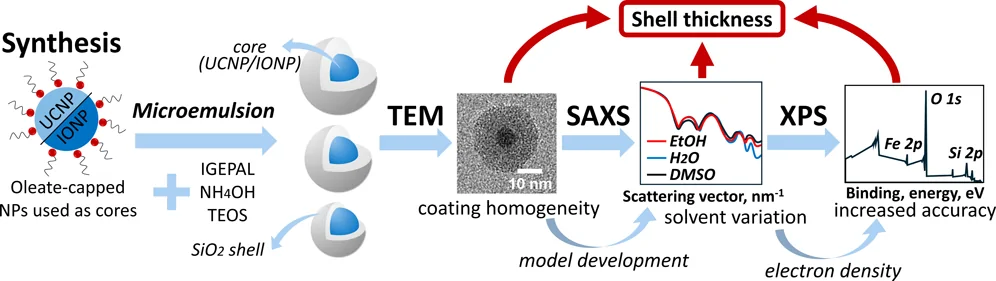
Schematic overview of the study showing the preparation of the IONP@SiO_2_ and UCNP@SiO_2_ core–shell particles from the initially synthesized oleate-capped IONP and UCNP cores and their initial characterization by TEM (upper panel), followed by shell thickness measurements by SAXS and XPS (lower panel) including a SAXS contrast variation experiment for refinement of the XPS data evaluation.

The TEM images shown in Figure 2 confirm the formation of silica shells around monodisperse 8.1 nm IONP and 8.8 nm UCNP cores, yielding spherical and concentric core–shell nanoparticles. Careful adaptation of the synthesis conditions permitted an increase in the shell thickness in steps of 2 nm to 4 nm, providing batches of samples with three different shell thicknesses for both core materials. Area equivalent circular diameters were determined (i) for the core particles before silica coating (see representative TEM images in Figure S1 in the Supporting Information (SI)) and (ii) for the complete core–shell particles. The difference between values (ii) and (i) is given as shell thickness in Figure 2. Image analysis was complicated by the tendency of nanoparticles with silica shells to agglomerate on the TEM grid during sample preparation, limiting the number of isolated core–shell particles that could be analyzed to less than 30 particles per image and impeding automatic image evaluation. The boundary between the core and shell cannot be clearly distinguished in the electron micrographs, especially for the thinnest shells. Slight variations in the coating thickness can be seen for the samples with a shell thickness below 5 nm. For sample IONP@SiO_2_–C, the complete coverage with a homogeneous silica shell could not be confirmed.

**Figure 2:**
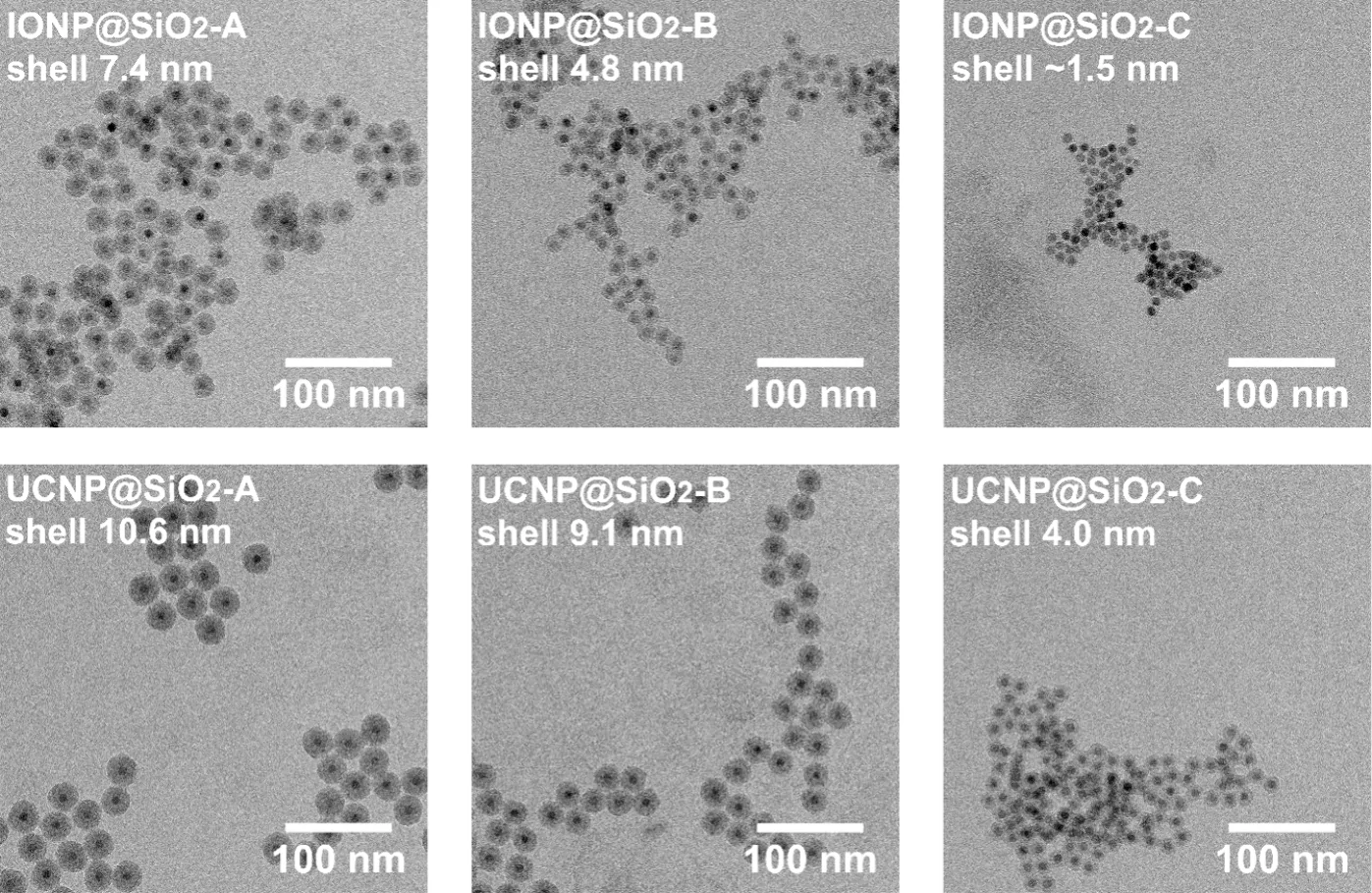
Transmission electron micrographs of the six IONP@SiO_2_ and UCNP@SiO_2_ core–shell nanoparticles. The shell thickness was derived from the differences of the equivalent circular diameters of the respective core–shell particles and the core particles measured prior to silica shelling.

SAXS was used to determine shell thickness values and uncertainties for each particle population in dispersion. The SAXS analysis employs a form-factor model that can be developed based on TEM images. Therefore, the data of all six core–shell particles was fitted with an ideally concentric and spherical core–shell form factor (SI, Figure S2) with independent distributions of shell thickness (log-normal) and core radius (Gauss) (see SI for details). The uncertainties of the data analysis were determined by χ^2^-scans of individual parameters.^17^ The diameter of the unshelled cores, which amounted to (8.71 ± 0.12) nm and (9.25 ± 0.12) nm for the IONPS and UCNPs, respectively, were fixed during the analysis of the core–shell particles. The shell thickness values derived from the SAXS data analysis (Table 1 and Figure 5) match the TEM results within the uncertainty of SAXS and the standard deviation of TEM.

**Table 1:** Core Diameter and Shell Thickness Obtained by TEM, SAXS, and XPS/HAXPES Together with Standard Deviations for Particle Size Distribution (TEM) and Standard Uncertainties for SAXS and XPS Data^a^

	Core diameter (nm)		Shell thickness (nm)					
Sample ID	TEM^a^	SAXS^b^	TEM^a^	SAXS	XPS^c^	HAXPES^c^	XPS^d^	HAXPES^d^
IONP@SiO2-A	8.1 ± 0.8	8.71 ± 0.12	7.4 ± 0.7	7.7 ± 0.4	5.4 ± 1.6	5.5 ± 1.7	5.9 ± 1.8	6.0 ± 1.8
IONP@SiO2-B			4.8 ± 0.5	4.4 ± 0.3	3.7 ± 1.1	3.6 ± 1.1	4.0 ± 1.2	4.0 ± 1.2
IONP@SiO2-C			1.5 ± 0.6	1.3 ± 0.9	0.6 ± 0.2	0.7 ± 0.2	0.7 ± 0.2	0.8 ± 0.3
UCNP@SiO2-A	8.8 ± 0.7	9.25 ± 0.12	10.6 ± 0.5	11.1 ± 0.3	6.4 ± 1.9	7.1 ± 2.1	7.0 ± 2.1	7.8 ± 2.4
UCNP@SiO2-B			9.1 ± 0.6	8.9 ± 0.3	3.7 ± 1.1	6.2 ± 1.9	4.0 ± 1.2	6.8 ± 2.1
UCNP@SiO2-C			4.0 ± 0.5	3.4 ± 0.3	2.1 ± 0.6	2.7 ± 0.8	2.4 ± 0.7	3.0 ± 0.9

a XPS and HAXPES results were calculated from (i) the mass density of bulk silica taken from the literature and (ii) the experimentally derived densities from the SAXS contrast variation experiment.

b Standard deviation of all single particles is given as a measure for the particle size distribution (PSD), not as uncertainty of the size measurement.

c For the SAXS measurements of the core diameter, two decimal places were provided, as this value was used for the calculations of the shell thickness. For the SAXS shell thickness measurements, one decimal place was given, consistent with the other measurement methods.

d Bulk density values in g cm^–3^: UCNP (Na_1.5_Y_1.5_F_6_) 4.30,^18^ IONP (Fe_3_O_4_) 5.17,^19^ SiO_2_ 2.20.^19^

e Experimental density values in g cm^–3^: UCNP 4.52; SiO_2_ 1.9; (IONP: no experimental value).

Subsequently, SAXS measurements were performed to determine the electron density of the particle materials as input for the later XPS and HAXPES analyses. For the mass density of silica materials, a literature value of bulk vitreous silica of 2.20 g cm^–3^ is typically used.^19^ This value likely differs from values of silica nanoparticles and shells synthesized by hydrolysis of organic silica precursors because the exact conditions of the particle fabrication and postsynthetic treatment define the final degree of silica network condensation, particle porosity, and size and can hence strongly influence mass density. Experimental values for colloidal silica range from 1.8 g cm^–3^ to about 2.3 g cm^–3^.^20^ To this end, two core–shell samples, UCNP@SiO_2_-A and -B, were measured by SAXS in various liquid mixtures with differing densities to vary the electron density contrast. Silica materials need polar liquids to form colloidally stable dispersions. Therefore, nine mixtures of dimethyl sulfoxide (DMSO), water, and ethanol with defined electron density were prepared under gravimetric control (SI, Table S1) and used for particle redispersion. The scattering curves were simultaneously fitted with a core–shell form-factor using the liquids’ electron density as input parameter, assuming an equal core radius and shell thickness distribution. Figure 3 shows example scattering curves for UCNP@SiO_2_-A, demonstrating a shift in the position and relative intensity of the scattering features as a function of the liquid’s electron density. The electron density of core and shell obtained from the fit parameters (Figure 3) match well for both samples and result in lower values than the literature bulk density for vitreous silica and agree well with the values for small colloidal silica particles reported by van Helden or Bogush et al.^21^

**Figure 3:**
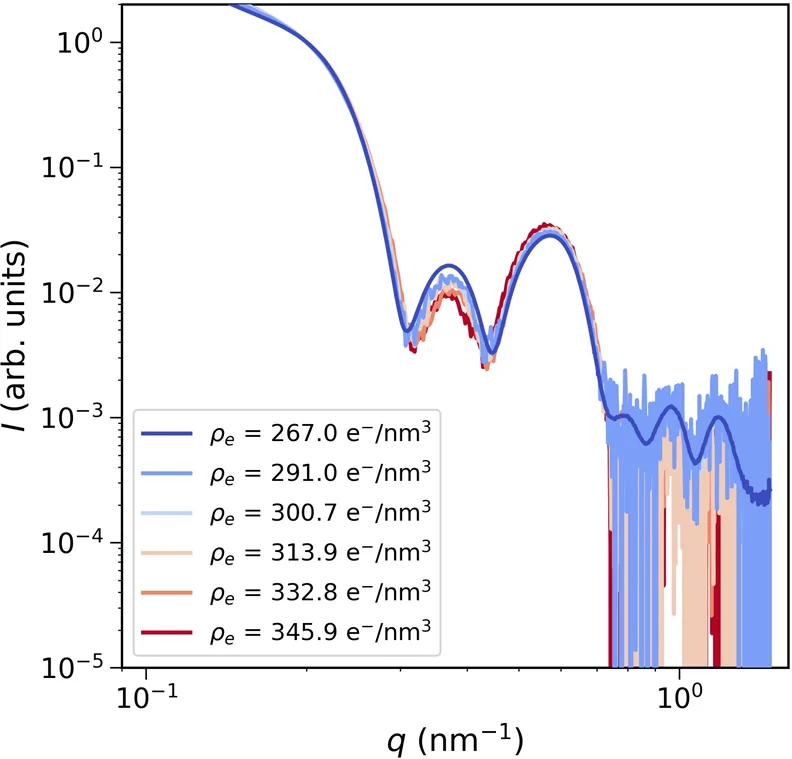
SAXS scattering curves of UCNP@SiO_2_-A measured in different liquid mixtures to vary the electron density contrast (left panel). Electron densities of core and shell for UCNP@SiO_2_-A and -B obtained from the fitting of the scattering curves (right table). The uncertainties in the electron densities were determined to the 1-sigma level, i.e., k = 1, using Gaussian error propagation.

The average of both values (1.9 g cm^–3^) was then used for the calculation of the shell thicknesses from XPS and HAXPES measurements in comparison with the literature bulk value of 2.2 g cm^–3^. Survey spectra of the six core–shell samples deposited onto gold-coated silicon wafers used for the XPS elemental quantification are provided in the SI (Figure S9 (XPS), Figure S10 (HAXPES)), along with the homogeneous-equivalent atomic fractions (expressed in atomic %) in Tables S2 and S3, respectively. For the thickness calculations, the most intense signals from the core or shell were used: for the IONP particles, the iron and silicon signals, and for the UCNP particles, the fluorine and silicon signals, respectively. The shell thicknesses given in Table 1 were then calculated by applying the method described by Shard et al. to the XPS and HAXPES data.^8^ The XPS method used for shell thickness calculation relies on the determination of the attenuation lengths of the photoelectrons through the core and shell materials. These properties can be estimated from the electron kinetic energy and average atomic number of the material through which the photoelectrons are traveling or more accurately if other material properties such as electron or mass density are known.^22^ The uncertainty of the XPS/HAXPES method is estimated to be 30 %.^23^ Major uncertainty contributions arise from (i) the uncertainty in the background subtraction procedure and possible exclusion of shakeup intensity in the peak and (ii) the uncertainty in the calculated sensitivity factors, primarily due to uncertainty in the theoretical photoionization cross sections and in the inelastic mean free path of electrons.

To highlight the importance of an accurate knowledge of the density of the shell material for XPS/HAXPES shell thickness measurements, in Figure 4, the XPS and HAXPES data, are plotted against the shell thicknesses measured by SAXS, using either the assumed bulk density of silica (2.2 g cm^–3^, data in black) or the experimentally derived silica density (1.9 g cm^–3^, data in red) for the respective calculations. A noticeably improved agreement with SAXS and TEM is obtained when the experimental density is used as an input, with the respective data (red data points) following a more consistent linear trend against the SAXS thickness. Likewise, there is a significant difference between the XPS- and HAXPES-calculated thicknesses, with the HAXPES values showing smaller variations from the linear trend. This effect is ascribed to the reduced surface sensitivity in HAXPES analysis, mitigating a degree of unevenness in the shell thickness. Please note that XPS and HAXPES will underestimate the shell thickness for nonuniformities in the shell thickness across the particle population.

**Figure 4:**
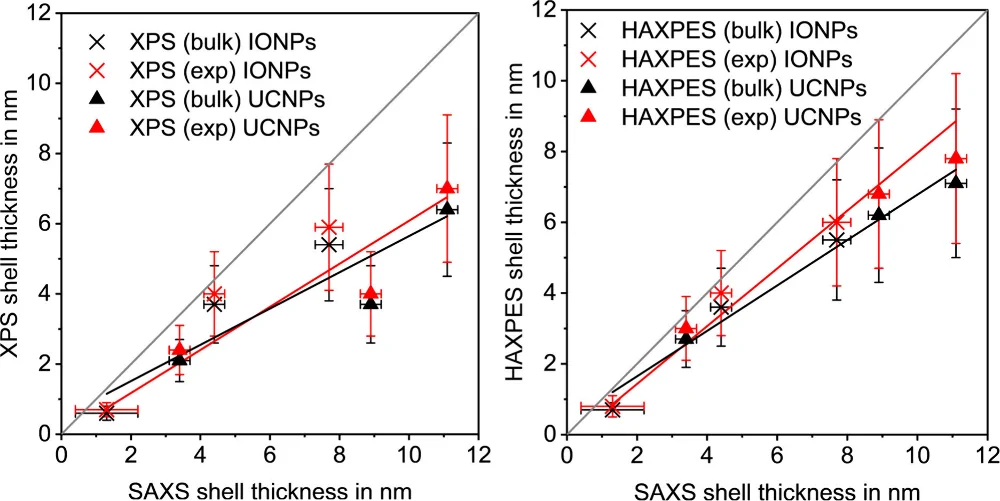
Shell thickness determined by XPS (left) and HAXPES (right) vs shell thickness by SAXS. Data evaluation with the bulk densities and the experimentally derived silica density are shown as black and red data points. The red and black lines are simple trend lines. The gray line corresponds to a perfect agreement of SAXS and XPS values (*x* = *y*).

XPS and HAXPES analysis using the experimentally derived density values results in slightly larger values for the thickness of the silica shells, which better match with the SAXS and TEM data (Table 1, last two columns, and Figure 5). For all six samples, the shell thickness values determined by XPS and HAXPES are consistently smaller than the shell thickness data provided by SAXS or TEM (Table 1 and Figure 5). However, the HAXPES values using the experimentally determined density values agree within the standard uncertainties with SAXS and TEM data, apart from the thickest shelled sample UCNP@SiO_2_-A sample, where the measurements agree only within the expanded uncertainties. A systematic underestimation of shell thickness using XPS and HAXPES compared to that of other techniques is consistent with this behavior. The exponential nature of photoelectron attenuation through a nonuniform overlayer results in underestimated thickness measurements, with the degree of deviation correlating with the variance in shell thickness and inversely correlating with the electron attenuation length. This effect is thus influenced by deviations across individual particles, e.g., due to noncentric cores and/or uneven coatings, and the dispersity of coating thickness across the particle population. Because of the inverse correlation with electron attenuation length, HAXPES is less strongly influenced by this effect. For our case of idealized modeling of SAXS and HAXPES/XPS measurements assuming concentric perfect spheres and concentric shells without impurities and core–shell intermixing, this can therefore cause systematic deviations between the values. Higher energy HAXPES sources, such as sources using Cr or Ga anodes, or synchrotron beamlines would likewise see a further reduction in this effect, corresponding to the increased electron energy and therefore higher attenuation lengths.

**Figure 5:**
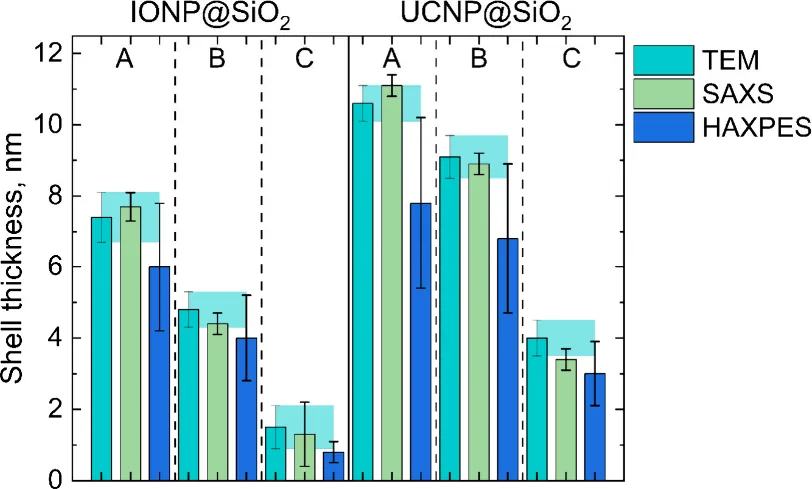
Comparison of the shell thickness values derived by TEM, SAXS, and HAXPES including shell thickness distribution for TEM shown by the light blue areas and standard uncertainties for SAXS and HAXPES.

To overcome limitations and sources of systematic variation of single-particle-based imaging and ensemble methods for characterizing advanced nanomaterials such as core–shell nanostructures, which often suffer from either poor statistics or the mandatory knowledge of material-specific parameters such as particle size distribution, refractive index, or density, complementary, advanced characterization methods should be used to accurately determine required input parameters at the nanoscale. Thereby, the common usage of bulk material properties can be circumvented for quantities that can be affected by material size and preparation method used. For properties that are difficult to measure in nanoscale materials such as mass or electron density and refractive index, novel approaches must be developed such as the presented contrast variation SAXS method, which simultaneously evaluates multiple scattering curves to determine the density of the core and shell. Use of the experimentally determined mass density of the silica shell material together with higher photon energy measurements for HAXPES compared to conventional XPS led to a significantly improved accuracy of shell thickness measurements for silica coatings with thicknesses of up to 10 nm. The remaining disparity between TEM- and HAXPES-measured thicknesses presents an indicator of the level of variation in the shell thickness, either on a population (polydispersity degree) or individual particle (coating inhomogeneity or unevenness) level. Direct measurements of the core and shell density by contrast variation SAXS also capture more accurately the material properties of the sample. Additional HAXPES measurements using higher energy X-rays, employing, e.g., a variable energy synchrotron source, could enable a further refinement of these measurements. By taking multiple measurements at increasing information depths, the estimated shell thicknesses are expected to approach an average value, validating the variation in shell thickness and providing a qualitative depth profile. Overall, our results underline the importance of a multimethod-based refinement for reliable and comparable shell thickness measurements using complementary characterization methods and the need to measure and establish such data for frequently used shelling materials.

## Experimental Methods

### Synthesis of Silica Coated Iron Oxide and Upconversion Nanoparticles

Oleate-capped IONPs (Fe_3_O_4_/Fe_2_O_3_) used as cores were synthesized following the two-step procedure from Park et al.,^24^ exploiting the thermal decomposition of iron oleate in 1-octadecene with oleic acid at 320 °C under an argon flow. Oleate-capped UCNPs (NaY_0.5_Gd_0.3_F_4_:Yb (18 mol %), Er (2 mol %)) used as cores were obtained using the high temperature method described by Wang et al.^25^ IONPs and UCNPs cores were coated with thin silica shells according to a reversed microemulsion approach described in the literature.^16^ Silica shell thickness was varied by simultaneously adjusting the amounts of NH_4_OH and TEOS.

### Transmission Electron Microscopy (TEM)

TEM measurements were performed using a Talos F200S microscope (Thermo Fisher Scientific) with an accelerating voltage of the electron beam at 200 kV. The samples were prepared on carbon-coated copper grids (Plano GmbH) by drop-casting and drying overnight at room temperature. TEM micrographs were analyzed using the software ImageJ (version 1.54g), manually evaluating around 150 particles per sample for the nanoparticle area, except for the IONP@SiO_2_–C sample, where only 70 particles could be analyzed by lines drawn across the nanoparticles.

### Small-Angle X-ray Scattering (SAXS)

SAXS measurements were performed at the four-crystal monochromator beamline of the Physikalisch-Technische Bundesanstalt (PTB) at the synchrotron radiation source BESSY II in Berlin-Adlershof. All details regarding data acquisition and evaluation are provided in the Supporting Information.

### (Hard) X-ray Photoelectron Spectroscopy

XPS and HAXPES measurements were performed using an Axis Supra+ spectrometer (Kratos Analytical Ltd.). For XPS a monochromatic Al Kα X-ray source was used, providing a photon energy of 1486.6 eV, and operating at a power of 75 W (15 kV with 5 mA emission current) for the “UCNP” samples and 225 W (15 kV with 15 mA emission current) for the “IONP” samples. For HAXPES, a monochromatic silver Lα X-ray source was used, providing a photon energy of 2984.3 eV, and operating at a power of 450 W (15 kV with 30 mA emission current). Samples were prepared for XPS/HAXPES measurements by drop-casting the nanoparticles from solution onto gold-coated silicon wafers. Details are provided in the Supporting Information.^26^

## Supplementary Material



## Notes

Additional particle characterization and experimental details on SAXS, XPS, and HAXPES measurements (PDF)
