# Supplementary material for: Combining Small-Angle X‑ray Scattering with Photoelectron Spectroscopy to Increase the Accuracy of Silica Shell Thickness Measurements on Inorganic Nanocrystals
Source: J Phys Chem Lett. 2026 Sep 2;17(43):12248–55. doi: 10.1021/acs.jpclett.6c02495 (PMC13726459; doi:10.1021/acs.jpclett.6c02495)
Supplement: Supplementary file 1 [file jz6c02495_si_001.pdf]

Supporting Information: Combining small-angle X-ray scattering with photoelectron spectroscopy to increase the accuracy of silica shell thickness measurements on inorganic nanocrystals

Sarah-Luise Abram‡ [1], Anna Matiushkina‡ [1,2], David Cant[3], Nicholas Engel[4], Robin Schürmann[4], Jörg Radnik[5], Elina Andresen[1], Carsten Prinz[1], Christian Gollwitzer[4], Alexander Gordon Shard[3], and Ute Resch-Genger[1]*

‡Both authors contributed equally.

[1]Federal Institute for Material Research and Testing (BAM), Division 1.2 Biophotonics, Richard-Willstaetter Str. 11, 12489 Berlin, Germany

[2]Free University Berlin, Department of Biology, Chemistry, and Pharmacy, Arnimallee 22, 14195 Berlin, Germany

[3]National Physical Laboratory (NPL), Hampton Road, TW11 0LW Teddington, UK

[4]Physikalisch-Technische Bundesanstalt (PTB), Abbestr. 2-12, 10587 Berlin, Germany

[5]Federal Institute for Material Research and Testing (BAM), Division 6.1 Surface Analysis and Interfacial Chemistry, Unter den Eichen 44-46, 12203 Berlin, Germany

TEM

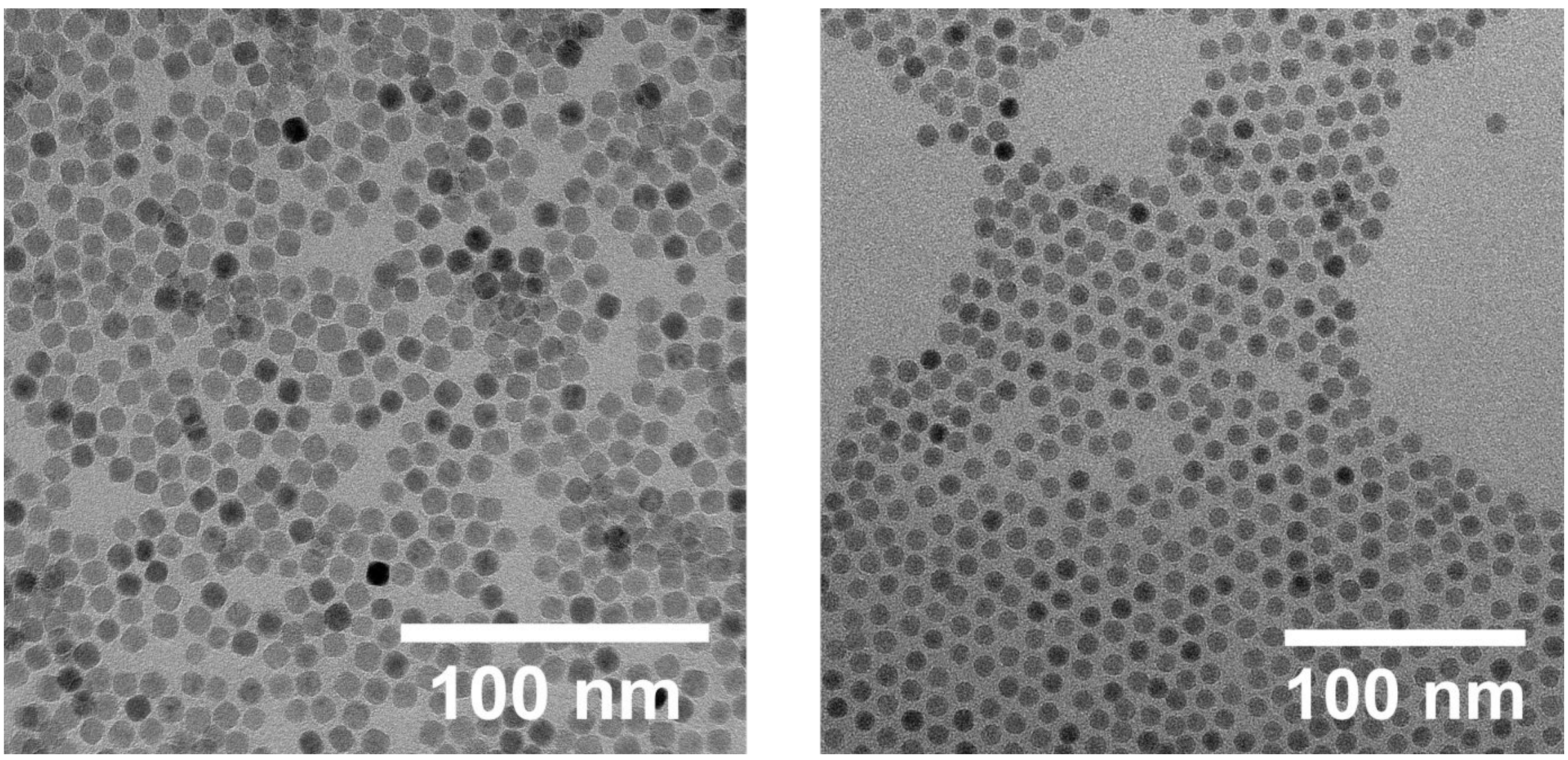

Figure S1: TEM images of iron oxide nanoparticles (IONPs, left) and upconversion nanoparticles (UCNPs, right) before silica coating. Images of the core particles were used to determine core size for the shell thickness calculation.

SAXS

Experimental setup

SAXS measurements were performed at the four-crystal monochromator beamline of the Physikalisch-Technische Bundesanstalt (PTB) at the synchrotron radiation source BESSY II in Berlin-Adlershof. SAXS measurements were performed at 8 keV with Si(111) monochromator crystals with a spectral resolving power of $E/\Delta E = 10^4$ and a photon flux in the range of $\Phi \simeq 10^9\ s^{-1}$. The X-ray beam has a cross-sectional area of approximately 150 µm x 400 µm (height x width) at the sample position. The SAXS data was recorded with an in-vacuum Pilatus 1M detector (Dectris Ltd, Baden, Switzerland) and WAXS with a Pilatus3 100k detector. Samples were measured in vacuum sealed, thin-walled glass-capillaries (WJM- Glas Müller) with a diameter of approximately 1 mm. To determine the background scattering from the solvent and the glass cuvettes, measurements of the buffer solution were performed in a separate cuvette. The optical path lengths through the individual capillaries (diameter of the capillary) were determined from transmission scans across the capillary. The exposure time for SAXS and WAXS measurements was set to 300 s. The sample detector distance of the SAXS detector was determined by the triangulation method and was found to be (2.8632 ± 0.0002) m. The distance of the WAXS detector was 205.997 mm determined from the 3rd d(001) long-period spacing peak of previously calibrated Ag-behenate. To calculate the 1-D scattering curve, the data from the 2 detectors were merged. AM60 (IONP@SiO_2-B) was merged at $q \approx 1.283$ nm^{-1}, AM61 (IONP@SiO_2-A) at $q \approx 1.30$ nm^{-1}, AM63 (UCNP@SiO_2-A) at $q \approx 1.16$ nm^{-1}, AM64 (UCNP@SiO_2-B) at $q \approx 1.32$ nm^{-1}, AM65 (IONP@SiO_2-C) at $q \approx 1.12$ nm^{-1}, and AM66 (UCNP@SiO_2-C) at $q \approx 1.28$ nm^{-1}. The WAXS data was normalized to the SAXS data, measured with a calibrated detector. Different normalization factors were allowed for data and background because the intensity was not normalized to the capillary thicknesses, that can vary slightly, prior to data merging. A mismatch between the SAXS and WAXS q-scales was corrected by adding an offset of 0.01 nm^{-1} to the WAXS q-scale.

For background subtraction, the SAXS data of ethanol was merged at the same q-values as the respective nanoparticle datasets. The background data was scaled to avoid oversubtraction. Again, since the capillary thickness can vary slightly, the scaling can vary. The scaling factors were: 0.975 for AM60 (IONP@SiO_2-B), 0.965 for AM61 (IONP@SiO_2-A), 1.0 for AM63 (UCNP@SiO_2-A), 0.96 for AM64 (UCNP@SiO_2-B), 0.95 for AM65 (IONP@SiO_2-C), 0.948 for AM66 (UCNP@SiO_2-C).

Data analysis

Fitting Model is using spherical form factor:

$$\mathrm{F_{sphere}(q,R)} = 3\,(\sin(\mathrm{qR}) - \mathrm{qR}\cos(\mathrm{qR}))/(\mathrm{qR})^3$$

The radii and volumes of core particle and core-shell particle are given by:

$$R_s = R_c + t,\ V_c = 4\pi R_c^3/3,\ V_s = 4\pi R_s^3/3$$

Thus, the scattering amplitude of core-shell particles is given by:

$$A_{\mathrm{cs}}(q;R_c,t) = (\rho_{\mathrm{core}} - \rho_{\mathrm{shell}})\,V_c\,F_{\mathrm{sphere}}(q,R_c) + (\rho_{\mathrm{shell}} - \rho_{\mathrm{solv}})\,V_s\,F_{\mathrm{sphere}}(q,R_s)$$

Gaussian shaped size distributions of core radius and lognormal distribution of shell thickness are assumed:

$$w_R(R) = \exp\left(-(R-R_{c0})^2/(2\sigma_{core}^2)\right),\ w_t(t) = \exp\left(-(t-t_0)^2/(2\sigma_{shell}^2)\right)$$

Leading to an expression for the scattering intensity:

$$I_{\mathrm{cs,pd}}(q) = \mathrm{scale} \iint w_R(R)\ w_t(t)\, A_{\mathrm{cs}}(q;R,t)^2\, \mathrm{d}R\, \mathrm{d}t + \mathrm{bkg}$$

Table S1: Gravimetric preparation of solvents and the calculated electron density using for the molecular weight M of Ethanol, H_2O and DMSO M_{EtOH}=46.068 g mol^{-1}, M_{H2O}=18.015 g mol^{-1}, M_{DMSO}=78.13 g mol^{-1}, for the respective number of electrons/molecule Z Z_{EtOH}=26 Z_H2O=10 Z_{DMSO}=44 and for the density @ 20˚C rho_EtOH=0.7857 g/mL, rho_H_2O=0.99565 g/mL, rho_DMSO=1.09144 g/mL.

m_{DMSO} / g	m_{H2O} / g	$m_{Ethanol}$ / g	ρ_e // nm^{-3}
pure			370.16
0.7912	1.3412		345.89
	pure		332.83
	0.1826	1.3811	273.26
	0.2696	1.3802	275.83
	0.6684	0.9183	291.04
	1.2250	0.9218	300.72
	1.43966	0.4602	313.86
		pure	267.04

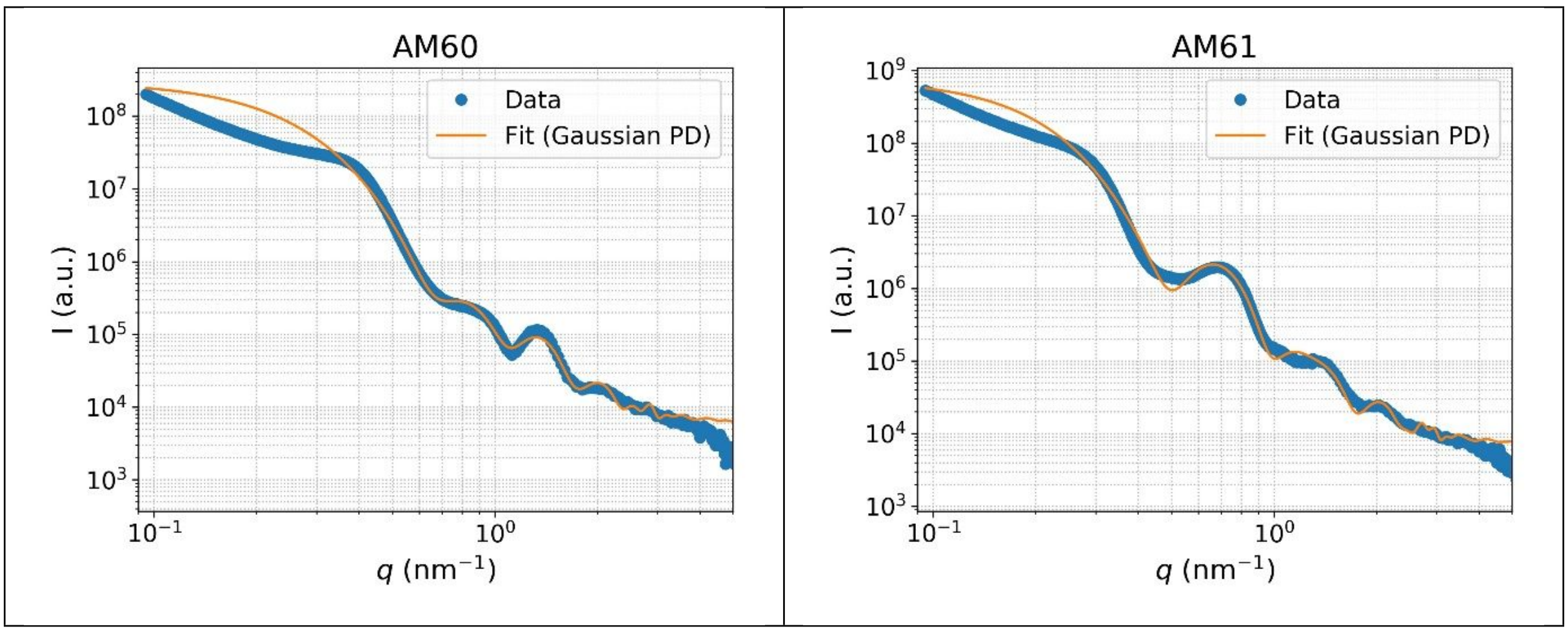

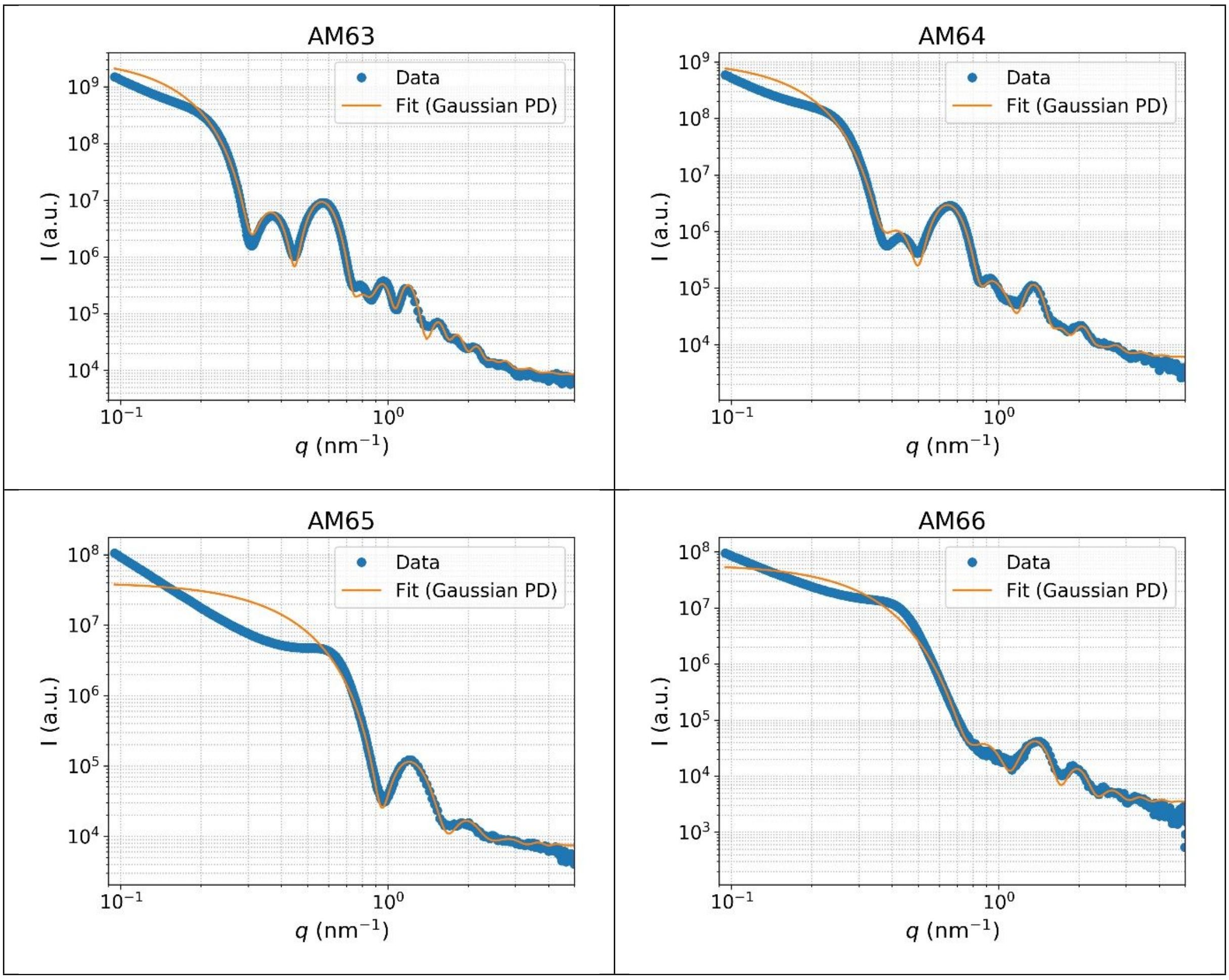

Figure S2: 1-dimensional scattering curves of AM60-66 with the core-shell form-factor fit. The data-range for the fit was selected from the first minimum to 4.2 nm^{-1} to avoid influence by the solvent peak. The samples AM61, 60 and 65 refer to the samples IONP@SiO_2-A, -B and -C whereas AM63, 64 and 66 refer to the samples UCNP@SiO_2-A, -B and -C.

UCNP@SiO_2-A and -B contrast variation

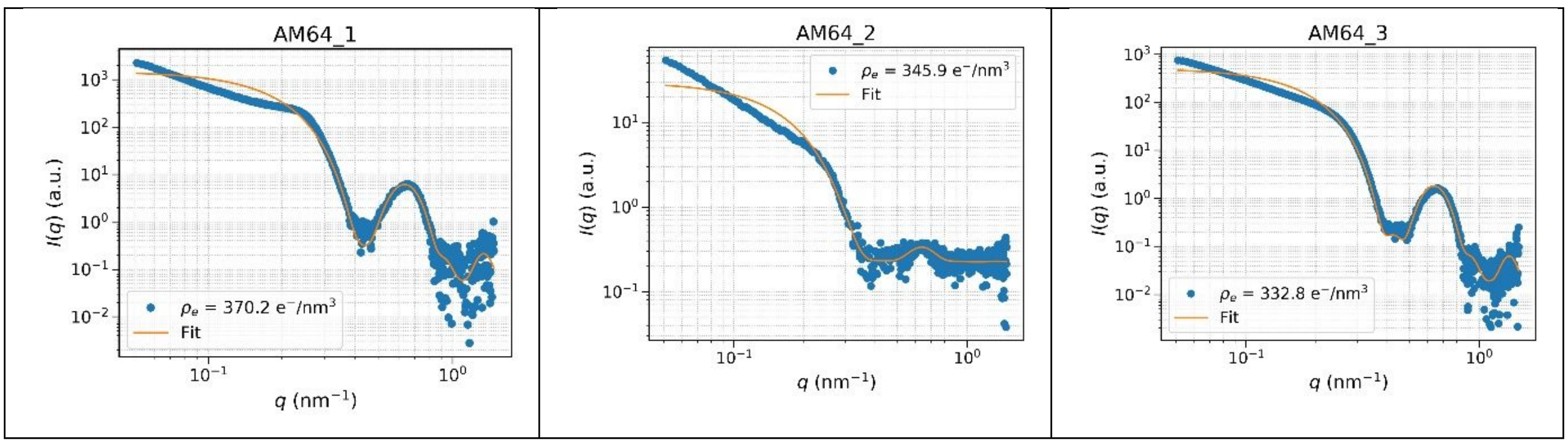

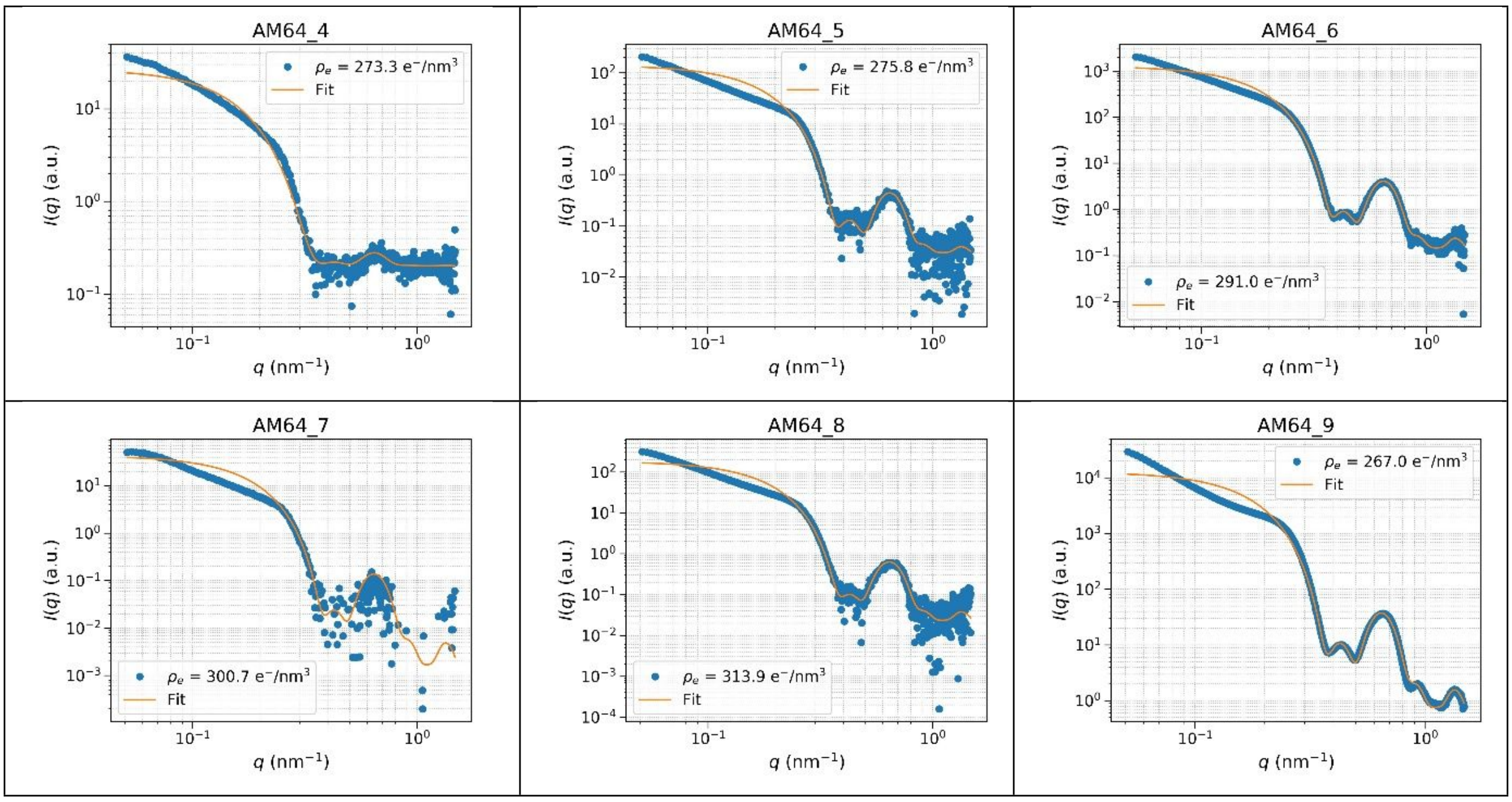

Figure S3: Simultaneous fit of UCNP@SiO_2-B in different solvent mixtures. Dimensional fitting parameters fixed to values shown in Table 1.

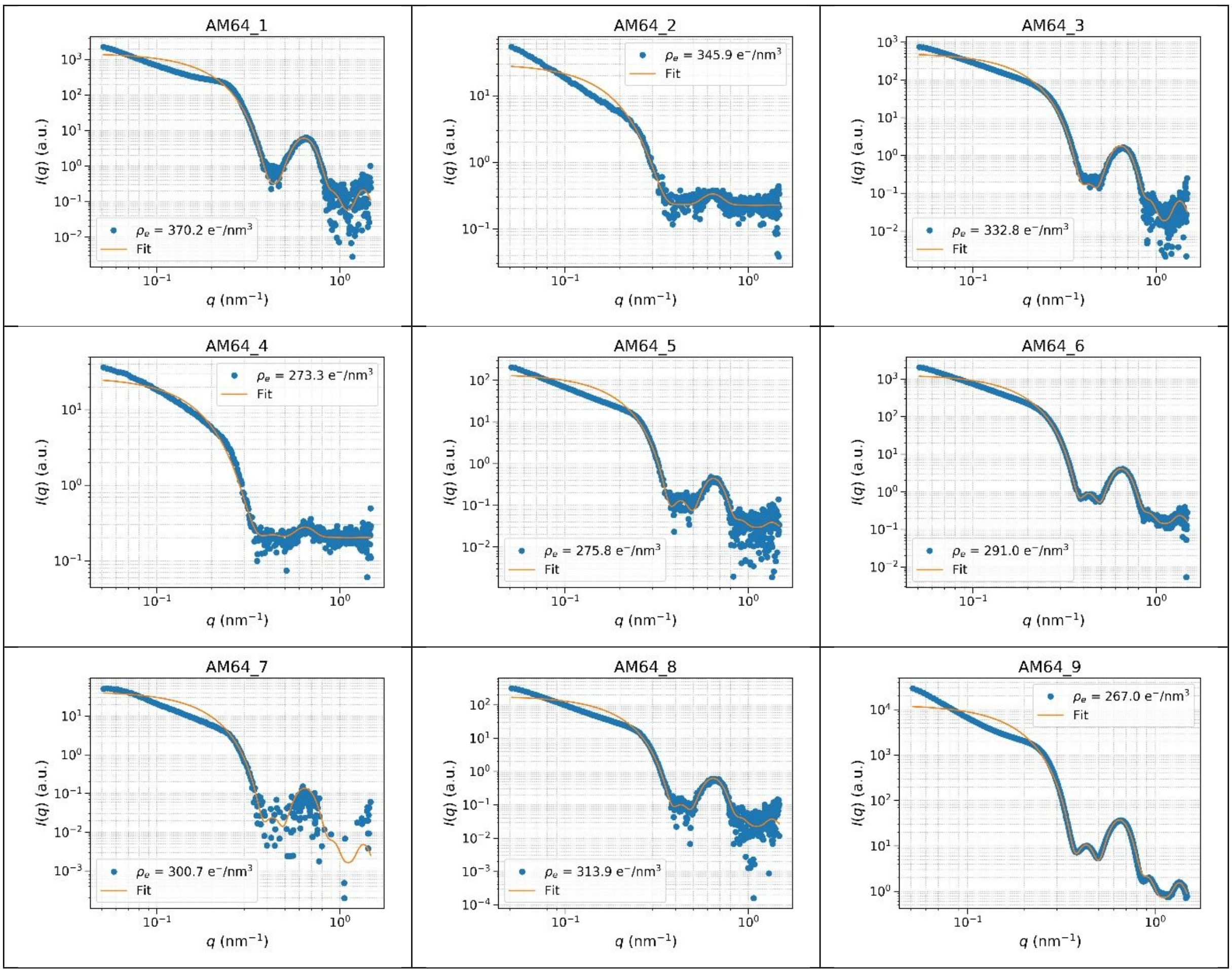

Figure S4: Simultaneous fit of UCNP@SiO_2-B in different solvent mixtures. Dimensional fitting parameters open.

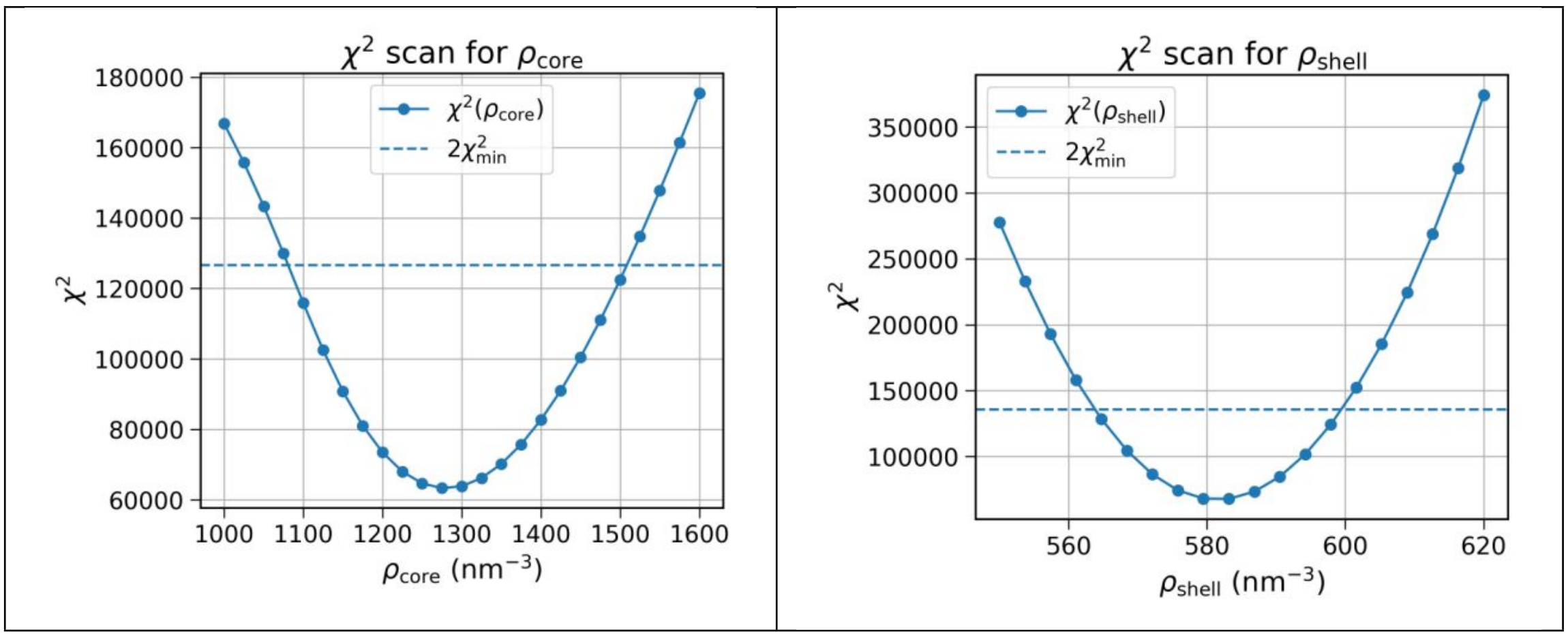

Figure S5: χ^2 for electron density ρ_e of core and shell of UCNP@SiO_2-B particles using fixed dimensional parameter for the size of the core.

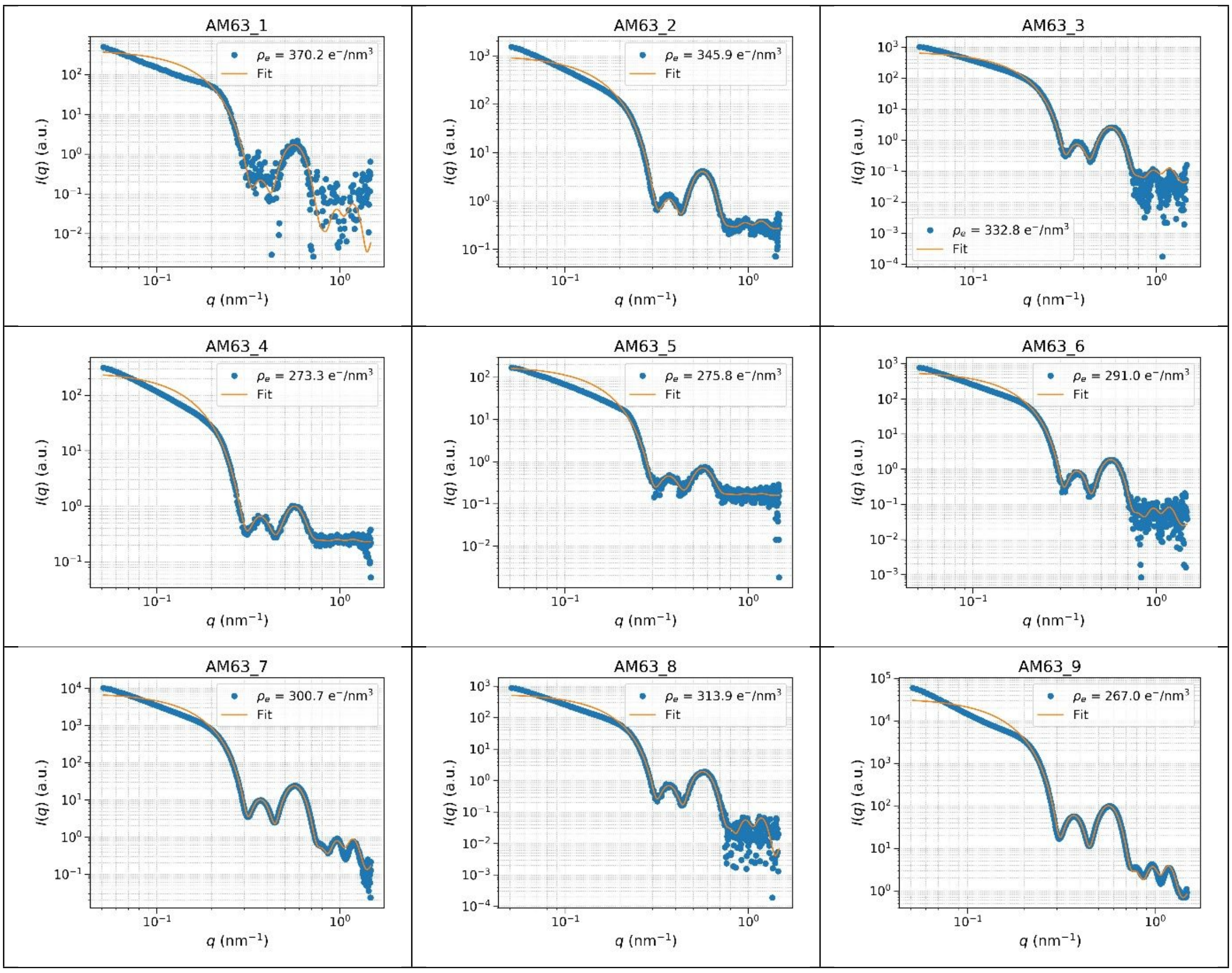

Figure S6: Simultaneous fit of UCNP@SiO_2-A in different solvent mixtures. Dimensional fitting parameters open.

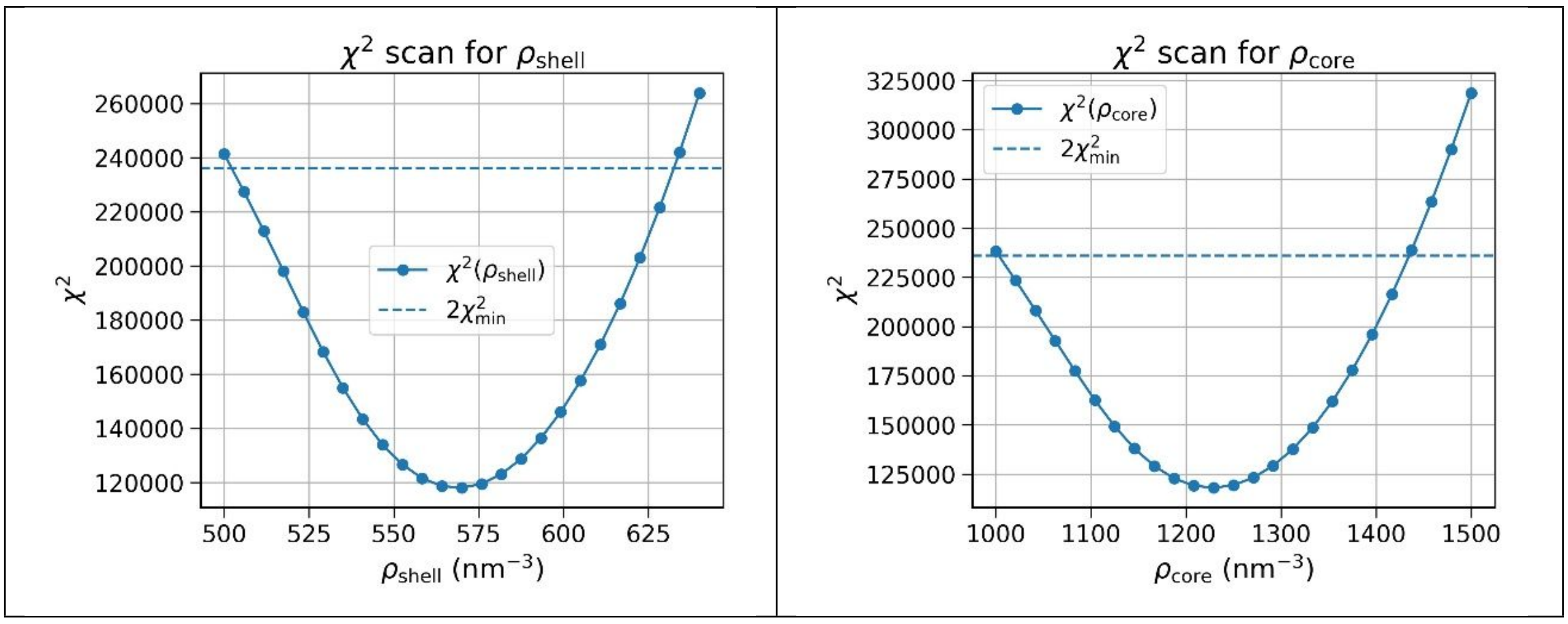

Figure S7: χ^2 for electron density ρ_e of core and shell of UCNP@SiO_2-A particles using fixed dimensional parameter for the size of the core.

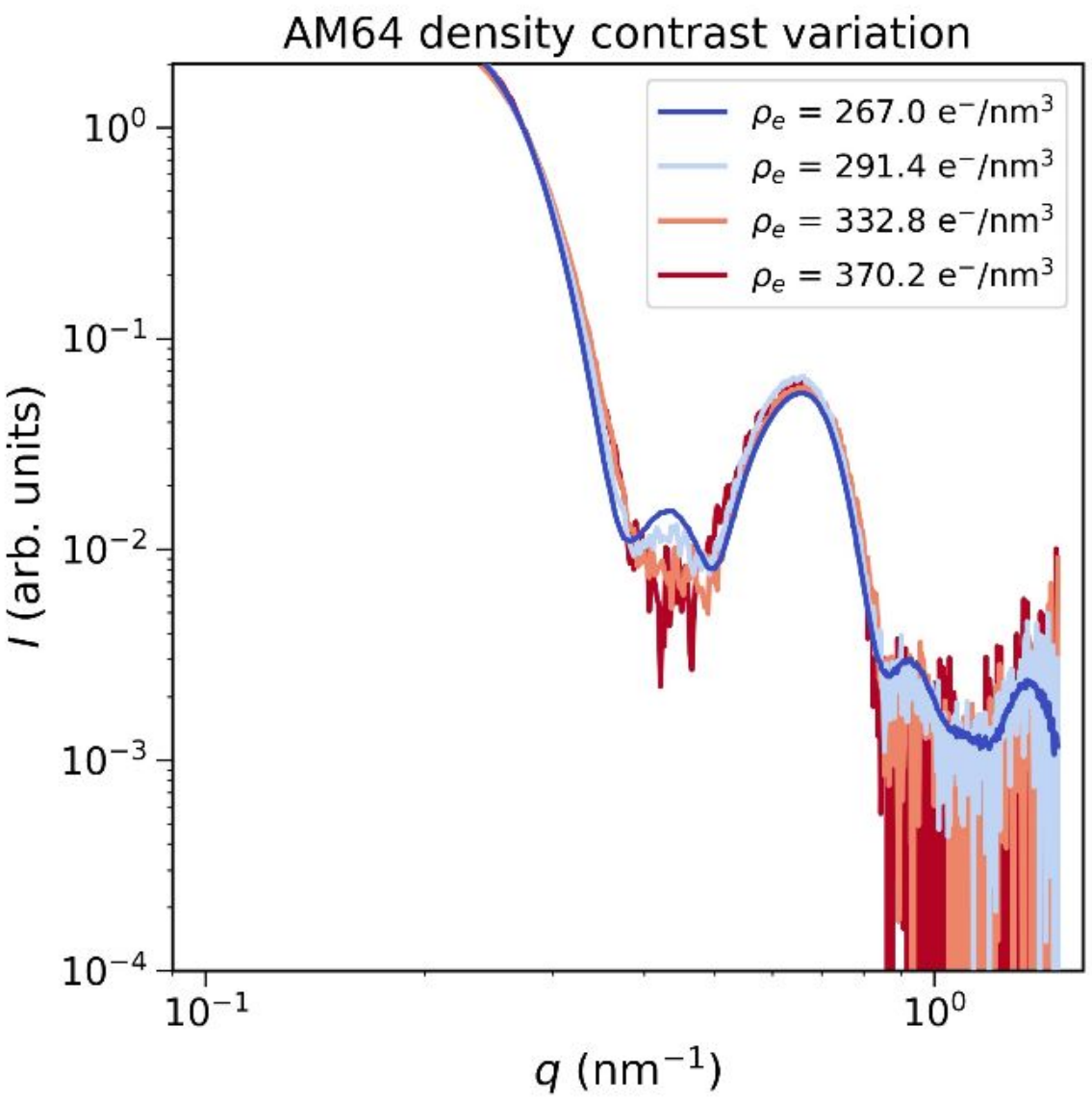

Figure S8: SAXS scattering curves of UCNP@SiO_2-B with different solvents.

XPS/HAXPES

Experimental

(Hard) X-ray photoelectron spectroscopy. XPS and HAXPES measurements were done with an Axis Supra+ spectrometer from Kratos Analytical Ltd. For XPS a monochromated aluminium Kα X-ray source was used, providing a photon energy of 1486.6 eV, and operating at a power of 75 W (15 kV with 5 mA emission current) for the 'UCNP' samples and 225 W (15 kV with 15 mA emission current) for the 'IONP' samples. For HAXPES, a monochromatic silver Lα x-ray source was used, providing a photon energy of 2984.3 eV, and operating at a power of 450 W (15 kV with 30 mA emission current). The analysis areas for both modes are about 300 μm x 700 μm. XPS survey spectra were acquired at a pass energy of 80 eV for the UCNP samples with the Hybrid lens mode which uses a magnetic collection lens, and 160 eV for the IONP samples using the electrostatic lens mode only. HAXPES survey spectra were acquired at a pass energy of 160 eV for both samples, using the 'Hybrid' lens mode for the UCNP samples and electrostatic mode for the IONP samples. Lens modes and pass energies were selected in order to optimize signal to noise ratios. Samples containing iron were analyzed without the use of the magnetic lens to avoid artefacts, however, the electrostatic lens mode results in significantly lower signal and hence, the use of higher X-ray source power and higher pass energies to maximize counts at the cost of energy resolution.

Samples were prepared for XPS/HAXPES measurements by drop-casting the nanoparticles from solution onto gold-coated silicon wafers in a vacuum desiccator. The deposition process was repeated with approximately 5-10 μl of solution per cycle until a visibly opaque deposit was observed, covering a large enough area of the substrate for XPS/HAXPES analysis. For this process, the vacuum desiccator is used without application of any sealing grease to prevent risk of surface contamination (e.g. from siloxanes in the grease) – the vacuum desiccator used for this is specifically set aside for surface analysis experiments and has not ever been greased.

XPS data were calibrated using the NPL transmission function calibration and were analyzed using the NPL Average Matrix Relative Sensitivity Factors (AMRSF's) for Al Kα X-rays.[1-3] HAXPES data were calibrated using a transmission calibration based on an extrapolation of the NPL method for Al Kα, and were analyzed using the NPL calculated HAXPES AMRSF's.[4, 5]

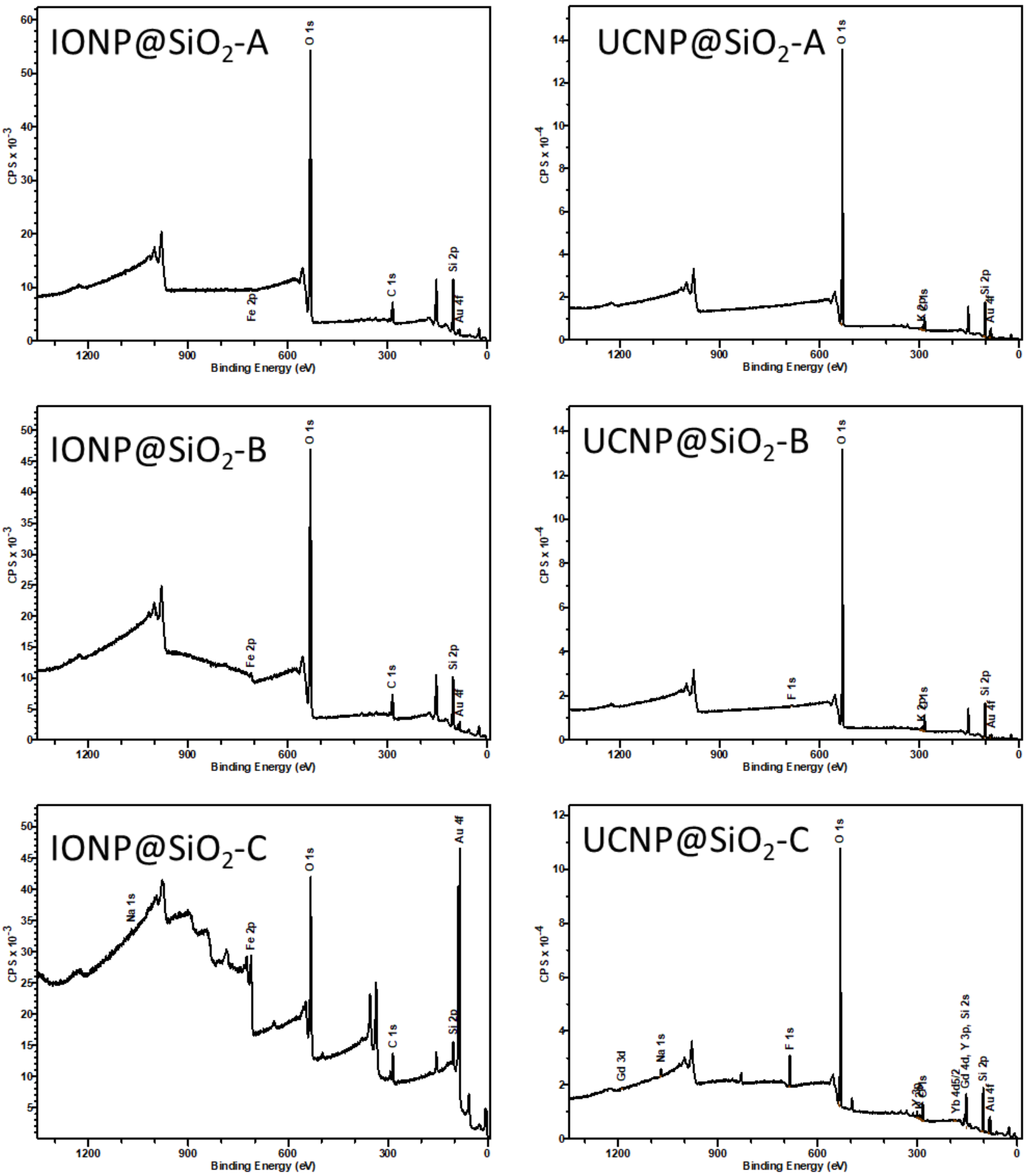

Figure S9: XPS survey spectra for all samples.

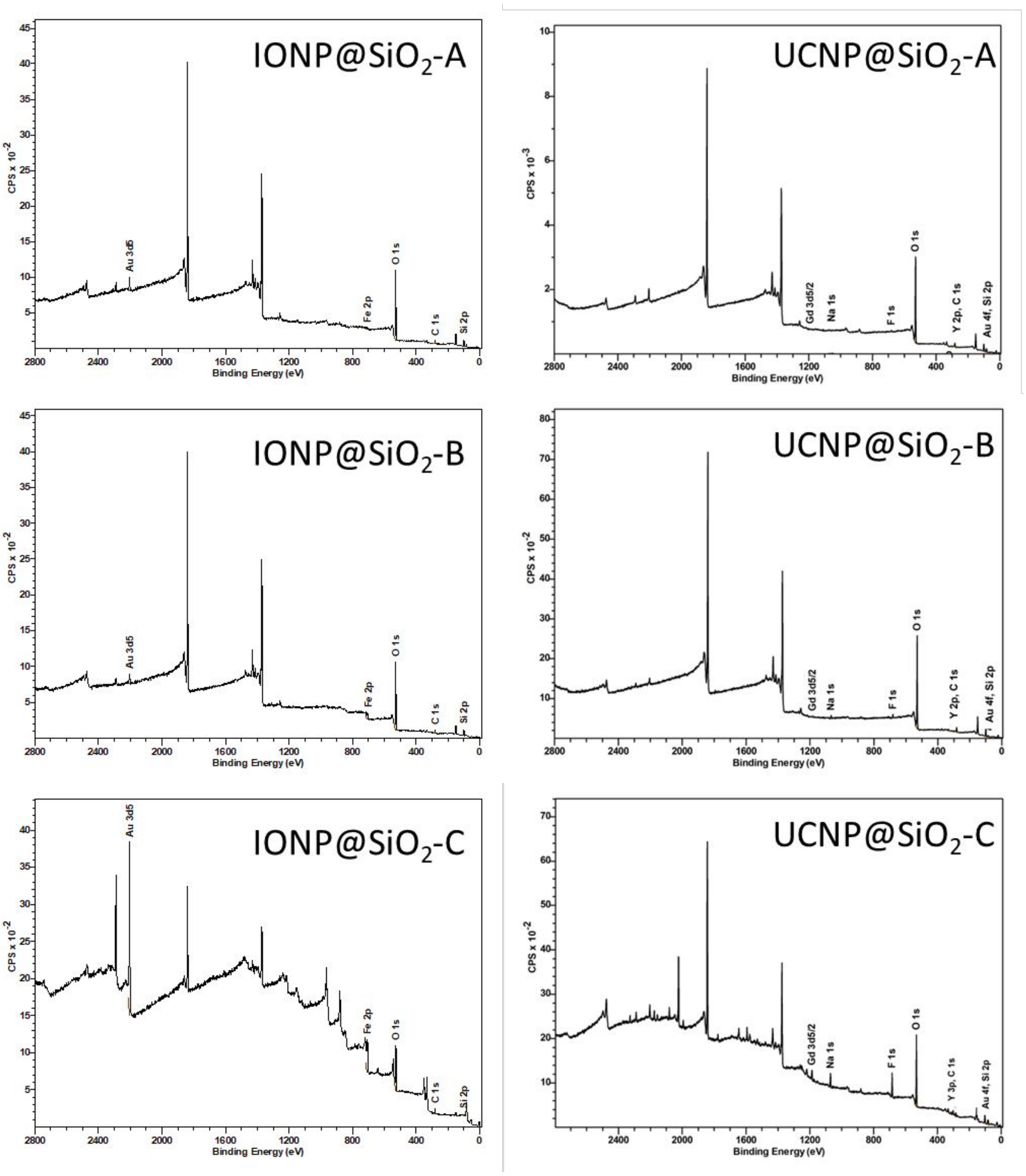

Figure S10: HAXPES survey spectra for all samples.

Table S2: XPS At%:

	Element and Peak Analysed									
	Au 4f	C 1s	F 1s	Gd 4d	K 2p	Na 1s	O 1s	Si 2p	Y 3p	Yb 4d5/2
UPNP@SiO_2-C	0.7	13.9	4.2	0.5	0.7	0.8	49.4	28.6	1.1	0.1
UPNP@SiO_2-B	0.2	14.1	0.3		0.6		53.8	31.1		
UPNP@SiO_2-A	0.5	12.5	0.1		0.5		55.1	31.3		

	Au 4f	C 1s	Fe 2p	K 2p	Na 1s	O 1s	Si 2p
IONP@SiO_2-C	9.1	16.6	14.4	1.6	0.7	47.7	9.9
IONP@SiO_2-B	0.6	14.4	0.7	0.9		56.1	27.4
IONP@SiO_2-A	0.4	12.6	0.2	0.7		56.4	29.8

Table S3: HAXPES At%:

	C 1s	Y 3p	O 1s	F 1s	Au 4f	Na 1s	Si 2p	Gd 3d5/2
UPNP@SiO_2-C	10.4	1.2	43.8	8.5	1.4	2.3	31.9	0.4
UPNP@SiO_2-B	10.5	0.3	53.1	1.3	0.4	0.3	34.2	0.0
UPNP@SiO_2-A	8.5	0.0	55.1	0.8	1.0	0.0	34.5	0.0

	O 1s	C 1s	Fe 2p	Au 3d5	Si 2p
IONP@SiO_2-C	55.2	21.1	13.2	3.2	7.3
IONP@SiO_2-B	55.1	18.6	2.9	0.2	23.1
IONP@SiO_2-A	57.7	13.2	1.2	0.3	27.6

(1) Seah, M. P. XPS reference procedure for the accurate intensity calibration of electron spectrometers— results of a BCR intercomparison co-sponsored by the VAMAS SCA TWA. *Surface and Interface Analysis* **1993**, *20* (3), 243–266. DOI: https://doi.org/10.1002/sia.740200309.
(2) Seah, M. P.; Gilmore, I. S.; Spencer, S. J. Quantitative XPS: I. Analysis of X-ray photoelectron intensities from elemental data in a digital photoelectron database. *Journal of Electron Spectroscopy*

and Related Phenomena **2001**, *120* (1), 93–111. DOI: https://doi.org/10.1016/S0368-2048(01)00311-5.
(3) Cant, David J. H.; Gorham, Justin M.; Clifford, Charles A.; Shard, Alexander G. Standard Approaches to XPS and AES Quantification—A Summary of ISO 18118:2024 on the Use of Relative Sensitivity Factors. *Surface and Interface Analysis* **2025**, *57* (2), 148–152. DOI: https://doi.org/10.1002/sia.7371.
(4) Shard, A. G.; Counsell, J. D. P.; Cant, D. J. H.; Smith, E. F.; Navabpour, P.; Zhang, X.; Blomfield, C. J. Intensity calibration and sensitivity factors for XPS instruments with monochromatic Ag Lα and Al Kα sources. *Surface and Interface Analysis* **2019**, *51* (7), 763–773. DOI: https://doi.org/10.1002/sia.6647.
(5) Cant, D. J. H.; Spencer, B. F.; Flavell, W. R.; Shard, A. G. Quantification of hard X-ray photoelectron spectroscopy: Calculating relative sensitivity factors for 1.5- to 10-keV photons in any instrument geometry. *Surface and Interface Analysis* **2022**, *54* (4), 442–454. DOI: https://doi.org/10.1002/sia.7059.
